# Intraoperative identification of a left non-recurrent laryngeal nerve by neuromonitoring: a critical anatomical finding—case report

**DOI:** 10.1093/jscr/rjag427

**Published:** 2026-06-03

**Authors:** Dioselina Lanzagorta Ortega, Ana Sofía Hernández Martínez, Juan Ramon Gomez Woodworth, Juan Francisco Peña Garcia, Moises Mercado Atri, Enrique Ricardo Jean Silver

**Affiliations:** Department of General Surgery, American British Cowdray Medical Center, Mexico City, Mexico; School of Medicine and Health Sciences, Tecnológico de Monterrey, Mexico City, Mexico; School of Medicine, Universidad Panamericana, Mexico City, Mexico; Department of General Surgery, American British Cowdray Medical Center, Mexico City, Mexico; Department of Internal Medicine and Endocrinology, American British Cowdray Medical Center, Mexico City, Mexico; Department of General Surgery, American British Cowdray Medical Center, Mexico City, Mexico

**Keywords:** non-recurrent laryngeal nerve, recurrent laryngeal nerve, intraoperative neuromonitoring, thyroid surgery, anatomical variation, laryngeal nerve injury

## Abstract

Injury to the recurrent laryngeal nerve (RLN) and its non-recurrent variant (NRLN) remains one of the most significant complications of thyroid and parathyroid surgery. The NRLN is a rare anatomical variation that arises directly from the cervical vagus nerve without looping into the thoracic cavity, most often associated with an aberrant subclavian artery. Identification of this structure is essential to avoid iatrogenic injury, particularly during thyroidectomy. We report the intraoperative neuromonitoring (IONM) identification of an unexpected non-recurrent left inferior laryngeal nerve; emerging directly from the vagus nerve and coursing horizontally towards the larynx. This case emphasizes the importance of IONM, surgical awareness of anatomical variation to prevent RLN and NRLN injury, and ensuring optimal postoperative laryngeal function.

## Introduction

The recurrent laryngeal nerve (RLN) is a critical neuroanatomical structure due to its close relationship with the thyroid gland and the larynx, as well as its essential role in phonation, swallowing, and airway protection. It originates from the vagus nerve (cranial nerve X), descends into the thorax, and then ascends toward the neck, looping under the subclavian artery on the right and beneath the aortic arch and ligamentum arteriosum on the left [1]. The RLN ascends within the tracheoesophageal groove and enters the larynx, providing motor innervation to all intrinsic laryngeal muscles except the cricothyroid and sensory innervation below the vocal folds.

Embryologically, the RLN derives from the sixth branchial arch. The caudal migration of the heart and regression of the distal aortic arches determine its recurrent course. When the right fourth aortic arch or the proximal right dorsal aorta fail to develop, the nerve does not descend into the thorax and instead arises directly from the cervical vagus nerve, resulting in a non-recurrent laryngeal nerve (NRLN) [1, 2]. This rare anatomical variant is most commonly associated with an aberrant right subclavian artery (ARSA), also known as arteria lusoria, which arises distal to the left subclavian artery and courses posterior to the esophagus [3]. Left-sided NRLN are exceptionally rare and may occur in the presence of a right-sided aortic arch, situs inversus, or dextrocardia [4].

Anatomically, the RLN runs in close proximity to the inferior thyroid artery (ITA) and the ligament of Berry, both key landmarks in thyroid surgery. In 61%–83% of cases, the nerve courses posterior to the ITA, while smaller proportions run anteriorly or between its branches [1]. Approximately 65%–79% of RLNs lie within the tracheoesophageal groove, although lateral or anterior positions increase surgical risk [5]. The incidence of right-sided NRLN ranges from 0.5% to 0.7%, whereas left-sided NRLN occur in ~0.04% of cases [6], with nearly 90% of right-sided variants associated with ARSA. Several classifications describe NRLN anatomy, including Toniato’s and Avisse’s systems, which are essential for surgical planning [6]. Additional RLN variations include extralaryngeal bifurcation, fan-shaped arborization, and variable relationships with the ITA [7].

## Case presentation

A 57-year-old woman with a history of hypothyroidism on levothyroxine therapy presented recurrent upper respiratory tract infections. Physical examination revealed mild asymmetric thyroid enlargement with a discrete left-sided palpable mass and no cervical lymphadenopathy. The patient showed no hoarseness or evidence of dysphonia, or other compressive symptoms such as dysphagia, or a foreign-body sensation.

**Figure 1:**
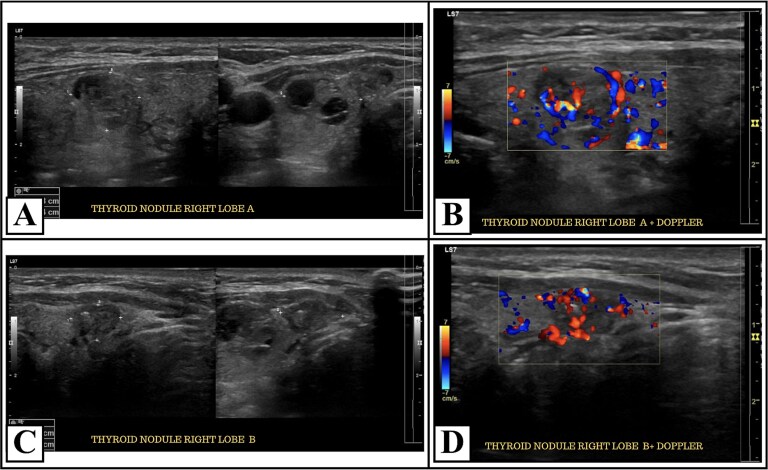
Thyroid ultrasound and doppler findings of right lobe nodules. Panel (A) shows a hypoechoic micronodule measuring 12 × 14 × 10 mm, with an irregular margin and a taller-than-wide configuration. Panel (B) shows color Doppler ultrasound demonstrating vascular flow within the hypoechoic right thyroid nodule. Panel (C) shows a solid hypoechoic nodule measuring 9 × 11 × 6 mm, with irregular margins and taller-than-wide shape. Panel (D) demonstrates color Doppler ultrasound of the solid right thyroid nodule; showing intranodular vascularity.

**Figure 2:**
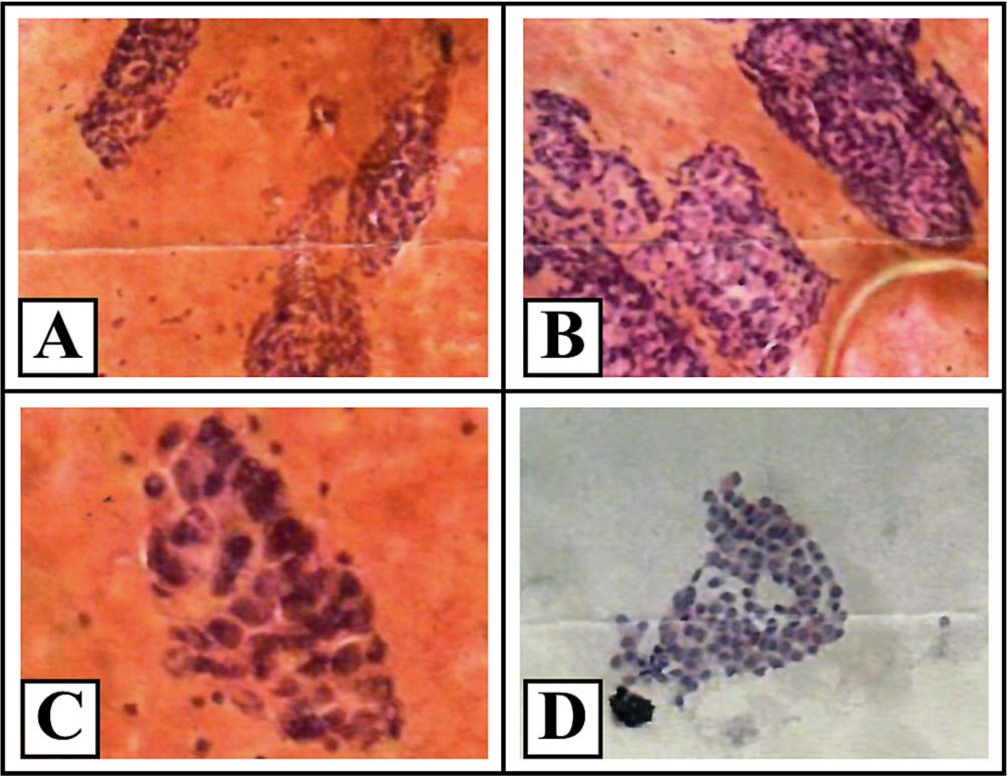
Cytopathological features consistent with Bethesda category VI (malignant). Representative cytological images from the right thyroid lobe stained with hematoxylin and eosin and Papanicolaou. The smears show papillary architectural patterns (A, ×200), marked cellular overlapping (B), irregular nuclei with loss of isodiametric shape (C, ×400), and fine, homogenous chromatin (D); consistent with Bethesda category VI (malignant).

A cervical ultrasound revealed two right thyroid nodules classified as TIRADS 4 and TIRADS 5 with hypoplasia of the left thyroid lobule (Fig. 1). No targeted assessment of the cervical vessels was done, and the ultrasound did not raise suspicion of an aberrant subclavian artery or any other vascular irregularities. Fine-needle aspiration biopsy of the right nodule reported papillary thyroid carcinoma (Bethesda VI) (Fig. 2). Thyroid function tests showed a TSH level of 3.84 mUI/L and a free T4 level of 1.09 ng/dL. Also, calcium levels were 9.17 mg/dL and PTH 23 pg/mL; both within range.

**Figure 3:**
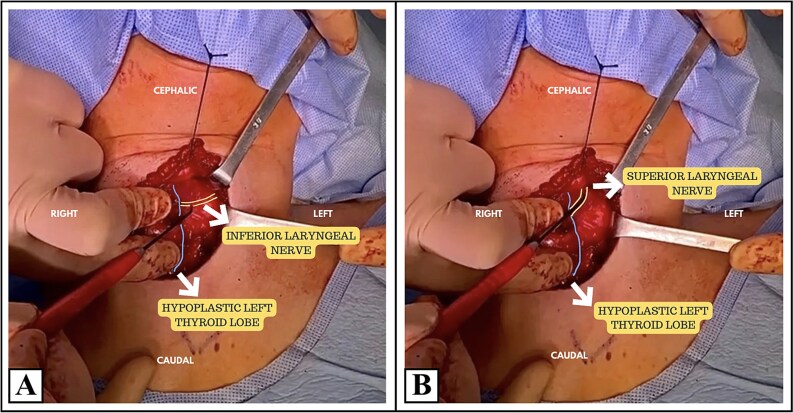
Intraoperative view of the thyroid surgical field. Image (A) represents the identification of the left inferior laryngeal nerve (non-recurrent course) using intraoperative neuromonitoring (IONM) in association with an hypoplastic left thyroid. Image (B) shows the left superior laryngeal nerve using IONM. These findings demonstrate an anatomical variation characterized by a non-recurrent left laryngeal nerve; confirmed through neuromonitoring signals rather than a recurrent course.

A total thyroidectomy with continuous neuromonitoring was performed. During dissection, the RLN was not found in its usual location, which prompted exploration of the vagus nerve. An exceptional anatomical finding was noted intraoperatively: a non-recurrent left inferior laryngeal nerve (Fig. 3). No postoperative vocal changes, symptoms of hypocalcemia or functional deficits were noted.

**Figure 4:**
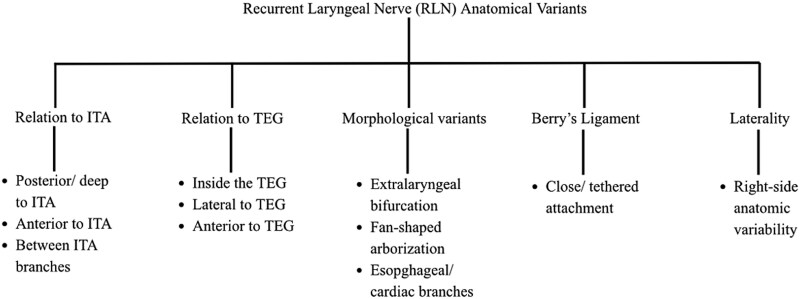
Representative hematoxylin and eosin-stained sections of the thyroid. Panel (A) shows an intrathyroidal papillary carcinoma focus, further demonstrating tumor multifocality. Panel (B) represents classical papillary architecture with fibrovascular cores and nuclear features consistent with conventional papillary thyroid carcinoma. Panel (C) demonstrates an additional intrathyroidal focus supporting multifocal disease. Finally, panel (D) shows microscopic extrathyroidal extension, with papillary carcinoma infiltrating adjacent extrathyroidal soft tissue. Images are shown at ×200 magnification.

Final histopathological evaluation confirmed conventional multifocal papillary thyroid carcinoma with microscopic extrathyroidal extension (Fig. 4). The patient subsequently received ablative therapy with 1850 MBq (50 mCi) of radioactive iodine. The patient expressed relief that her voice remained normal after surgery and was satisfied with the outcome.

## Discussion

The clinical manifestations related to the RLN or NRLN injury result in hoarseness, dysphonia, dysphagia, and glottic obstruction; secondary to unilateral or bilateral vocal cord paralysis [4]. Left-sided NRLNs are extremely uncommon, and most reports link them to vascular anomalies. When it is associated with an ARSA, patients may also experience dysphagia lusoria, which is characterized by esophageal compression symptoms such as progressive dysphagia, chronic cough, or weight loss. Preoperative identification of a NRLN is vital for minimizing surgical complications. The absence of the “Y-sign” (brachiocephalic trunk and its bifurcation) on ultrasonography suggest an ARSA and therefore a NRLN with 95% accuracy [2]. Computed tomography angiography or magnetic resonance imaging can confirm the aberrant subclavian course; while barium swallow reveals the characteristic “bayonet sign” [3]. The absence of preoperative suspicion makes this case particularly relevant for routine thyroid surgery.

**Figure 5:**
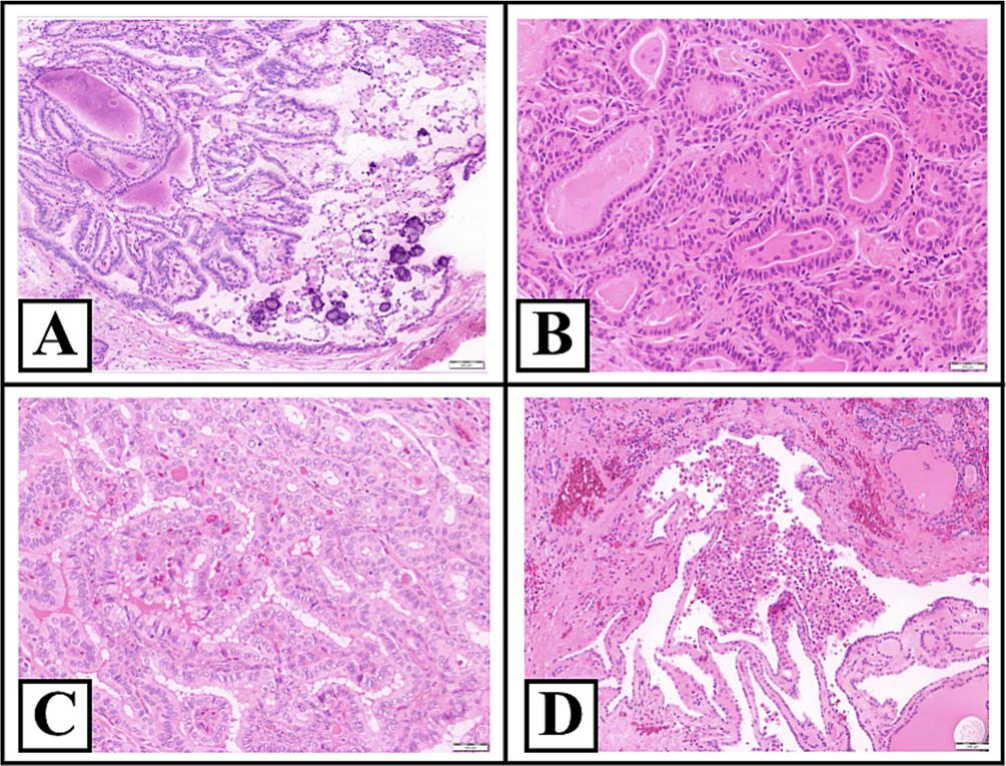
Recurrent laryngeal nerve (RLN) anatomical variants. Inferior thyroid artery (ITA). Tracheoesophageal groove (TEG).

Intraoperative neuromonitoring (IONM) is a valuable tool to identify and preserve the laryngeal nerves [8]. Beside meticulous dissection in the capsular plane, the gold standard of thyroid surgery remains the visual identification of the nerve before ligating vessels; especially the ITA (Fig. 5). Also, it is crucial a careful dissection around the ligament of Berry, where the RLN enters the larynx and therefore is a common site of injury [9]. If the nerve differs from its expected location, exploration of the vagus nerve is recommended. Advantages of IONM in this setting include improved nerve mapping, enhanced surgical safety, and increased confidence in identifying anatomical variations. Its limitations should also be acknowledged; including the possibility of false-negative or false positive-signals, dependence on proper electrode placement, technical expertise, and the fact that it does not replace careful operative technique. Unrecognized NRLN carry a sixfold higher risk of intraoperative injury compared to typical RLN; with reported rates of 13%–14% versus 2%–4% in standard anatomy [6]. Variations such as extralaryngeal branching or lateral displacement outside the tracheoesophageal groove carry a moderate risk but generally favorable outcomes when recognized early. However, damage to a non-recurrent form may cause permanent dysphonia because the nerve lacks the redundant loop that buffers mechanical stress [10]. Inversini *et al*. reported a right-sided NRLN identified using IONM; preventing inadvertent damage during thyroidectomy [10]. Therefore, a combined strategy of preoperative imaging, anatomical identification, and functional monitoring offers the preservation of nerve integrity with preoperative planning, and accurate dissection.

Injury to the RLN and its rarer variant NRLN remains one of the most significant complications of thyroid and parathyroid surgery [8]. Preventive measures in these scenarios include capsular dissection to preserve vascularization, minimal traction, and selective vascular ligation after nerve visualization [9]. IONM enables real-time identification and protection of the nerve through electromyographic feedback. The meta-analysis by So and Thompson compared surgical risk between recurrent and non-recurrent variants, confirming that NRLN are significantly more vulnerable if not recognized preoperatively [6]. Continuous monitoring allows immediate cessation of manipulation if the electromyographic amplitude decreases, reducing the risk of permanent injury as it has been demonstrated in a recent umbrella review [8]. Overuse of thermal sealing devices near the ligament of Berry should be avoided, as traction injuries and thermal damage are the most frequent causes of RLN dysfunction. Surgeons should apply minimal traction, maintain tissue hydration, and keep energy devices at least 5 mm away from the nerve. In patients known with vascular anomalies, it is needed a high-resolution ultrasonography as well as a lateral approach to the thyroid lobe, improving exposure of both the aberrant artery and the nerve. Thus, integrating these practices exemplify the anatomical science and surgical technology in preserving both laryngeal and thyroid function.

## Conclusions

The recurrent and non-recurrent laryngeal nerves are of major anatomical and clinical importance. This case is notable due to the unexpected intraoperative identification of a left-sided NRLN. Their embryologic development is closely linked to aortic arch morphogenesis, and their anatomical variations carry significant surgical implications. Preoperative imaging combined with intraoperative neuromonitoring represents the most effective strategy to prevent nerve injury. A thorough understanding of embryology, anatomy, and surgical technique is essential to preserve laryngeal function and optimize patient outcomes.
